# Difference in Intestinal Flora and Characteristics of Plasma Metabonomics in Pneumoconiosis Patients

**DOI:** 10.3390/metabo12100917

**Published:** 2022-09-28

**Authors:** Yingdi Li, Kun Xiao, Shuyu Xiao, Miaomiao Wang, Shasha Pei, Heliang Liu, Yuping Bai, Yulan Jin, Jinlong Li, Xiaoming Li, Qingan Xia, Fuhai Shen

**Affiliations:** 1 Hebei Province Key Laboratory of Occupational Health and Safety for Coal Industry, School of Public Health, North China University of Science and Technology, Tangshan 063210, China; 2 Tangshan Center of Disease Control and Prevention, Tangshan 063000, China; 3 Tangshan City Workers’ Hospital, Tangshan 063000, China

**Keywords:** pneumoconiosis, metabolomics, intestinal flora

## Abstract

From the two perspectives of intestinal flora and plasma metabolomics, the mechanism of occurrence and development of pneumoconiosis was explored to provide a new target for the prevention and treatment of pneumoconiosis. In this study, 16S ribosome DNA (16SrDNA) gene sequencing technology was used to analyze the differences in intestinal flora of each research group through operational taxonomic units (OUT) analysis, cluster analysis, principal component analysis (PCA), partial least square discriminant analysis (PLS-DA), Kyoto Encyclopedia of Genes and Genomes (KEGG), and other analytical methods were used to analyze the differences in plasma metabolites between the study groups. Metabonomics analysis showed that the plasma metabolites of pneumoconiosis patients were significantly different from those of normal people. Fold change > 2; vip > 1; *p* < 0.05 were the screening criteria. In the positive and negative mode, we screened ten types of differential metabolites. These ten metabolites were upregulated to varying degrees in the pneumoconiosis patients. Seven metabolic pathways were obtained by analyzing the metabolic pathways of different metabolites. Among them, the aminoacyl tRNA biosynthesis pathway changed most obviously. The α diversity of two groups of intestinal flora was analyzed using the 16SrDNA technique. The results showed that there was no significant difference in ACE, Chao1, Shannon, or Simpson in the two groups (*p* > 0.05). Beta diversity analysis showed that there were differences in microbial communities. In pneumoconiosis patients, the abundance of Prevotellaceae increased, and the other nine species decreased. Compared to the control group, the abundance of Prevotellaceae in the intestinal flora of pneumoconiosis increased, and the abundance of the other nine species decreased. Compared to controls, ten substances in the plasma metabolites of pneumoconiosis patients were upregulated. Seven metabolic pathways were obtained by analyzing the metabolic pathways of different metabolites. Among them, the aminoacyl tRNA biosynthesis pathway changed most significantly. This provided a theoretical basis for further study on the pathogenesis, early prevention, and treatment of pneumoconiosis.

## 1. Introduction

Pneumoconiosis is widely distributed all over the world. It is a chronic systemic disease caused by accumulation of dust in the body caused by workers’ long-term exposure to production dust [1,2]. The clinical symptoms of pneumoconiosis are almost the same as those of chronic obstructive pulmonary disease (COPD) [3], both of which are mainly confined to the respiratory system. However, some systemic symptoms may appear at the same time, such as weakened digestive function, poor gastric emptying, abdominal distension, and constipation.

Most pneumoconiosis patients died of complications, and there is no effective treatment method so far. At present, drugs and whole-lung lavage (WLL) are mainly used in clinics. Although they can effectively alleviate the development of pneumoconiosis, they also have some drawbacks. In this regard, the researchers put forward that while studying the pneumoconiosis itself, they should open up new ideas and strategies for prevention, diagnosis, treatment, and prognosis: applying the viewpoint of system biology, comprehensively understanding the functions and potential mechanisms of each system from the overall level, and finding prospective biomarkers in time so as to carry out intervention measures to slow down progress, reduce clinical symptoms, and improve treatment as soon as possible [4]. Studies have explored the changes in metabonomics in COPD, idiopathic pulmonary fibrosis (IPF), and asthma and found metabolic pathways related to different metabolites [5]. In addition, there are more than 10 trillion bacteria in the gastrointestinal tract, which comprise the second largest genome of human beings—intestinal flora. Many studies have confirmed that the occurrence and development of digestive tract tumors, nonalcoholic fatty liver, inflammatory enteritis, obesity, diabetes, and other diseases are closely related to the imbalance of intestinal flora. Therefore, exploring the relationship between the occurrence and development of host diseases and intestinal flora is of great importance for opening up new ideas of disease prevention and treatment.

Some studies have shown that with the aggravation of disease [6], pneumoconiosis patients’ lung function was damaged, lung parenchyma was destroyed, and lung blood vessels were abnormal. Repeated infection caused the damage of ventilation function, which caused the imbalance of pulmonary ventilation blood flow, hypoxemia, accelerated ATP degradation, and increased uric acid. At the same time, acid–base imbalance destroyed a large number of red blood cells, which also led to increased uric acid production. It has been found that respiratory diseases are closely related to serum 25-hydroxyvitamin D3 [7]. Pneumoconiosis easily affects the body’s endocrine system, causing disorder of calcium and phosphorus metabolism and inducing the decrease of 25-hydroxyvitamin D in serum. At the same time, pneumoconiosis patients are repeatedly infected, which induces congestion in the gastrointestinal tract, weakens the absorption capacity of vitamin D in the small intestine, and leads to the decrease of 25-hydroxyvitamin D in serum [8].

Intestinal diseases or other diseases caused by the change in intestinal microbial community structure. Moreover, many life activities in cells take place at the level of metabolites, such as cell-to-cell communication, energy transfer, and cell signal release, which are regulated by metabolites. The combination of metabonomics and the difference in intestinal flora can be widely used in the dynamic monitoring of metabolic substances and intestinal microflora composition in different disease groups so as to determine the specific metabolic changes and intestinal flora changes of specific diseases, which can be used as biomarkers in clinical disease diagnosis and for the specific diagnosis of diseases and the exploration of disease mechanisms, providing theoretical basis [9]. Therefore, based on research progress in China and abroad combined with the previous research results of pneumoconiosis proteomics in our research group, using 16SrDNA gene sequencing technology and metabonomics method comprehensively, and taking pneumoconiosis patients as the research object, this paper explores the pathophysiological change mechanism during the occurrence and development of pneumoconiosis from the perspectives of intestinal flora and plasma metabonomics and provides new insights for its prevention, diagnosis, treatment, and rehabilitation.

## 2. Materials and Methods

### 2.1. Research Object

We collected and sorted the data of newly admitted pneumoconiosis patients in the pneumoconiosis department of Beidaihe Coal Mine Workers’ Sanatorium. According to the inclusion criteria and exclusion criteria, 15 patients with coal worker’s pneumoconiosis stage I were selected as the study group, and their plasma samples were collected.

In the Physical Examination Center of Xing Tai Dong Pang Coal Mine Hospital, we collected and sorted the data of physical examination personnel. According to the inclusion criteria and exclusion criteria, 15 volunteers were selected as the control group. We then collected their plasma and fecal samples. Subjects did not smoke or drink.

Inclusion criteria of the study group: pneumoconiosis stage with a definite history of pneumoconiosis, agreed to participate in the research of this topic. Exclusion criteria: serious disease, antibiotics were used in the past 3 months.

Inclusion criteria of the control group: according to the principle of group matching, the age difference between the subjects in the study group and the control group was not more than 5 years old, the same gender. Subjects did not smoke or drink in either group. Exclusion criteria: antibiotics were used in recent 3 months.

### 2.2. Experimental Reagents and Instruments

Main reagents: methanol (Shenzhen City Life Technology Co., Ltd., CNW Technologies, Shenzhen, China), acetonitrile (Tianjin Da Mao Chemical Reagent Factory, CNW Technologies, Tianjin, China), ammonium acetate (Merda Technology Inc, Beijing, China), ammonia (Beijing Chemical Company, CNW Technologies, Beijing, China), fecal genomic DNA extraction kit (Beijing Tiangen Biochemical Technology Co., Ltd., Beijing, China), bacterial 16SrDNA V3-V4 universal primer (Shanghai Baiqu biology Co.,Ltd.), 50× TAE Buffer (Beijing solab Technology Co., Ltd., Beijing, China), green PCR Master Mix (2×) (Thermo Scientific, Waltham, MA, USA), nucleic acid DNA marker (100 bp) (Beijing solab Technology Co., Ltd., Beijing, China), nucleic acid dye II (biological), agarose (Shanghai Bay gene biotechnology company, Shanghai, China).

Instruments and equipment: ultra-performance liquid chromatography (Agilent), high resolution mass spectrometer (AB SCIEX), centrifuge (Thermo Fisher Scientific, Shanghai, China), balance (Sartorius), grinder (Shanghai Jingxin Technology Co., Ltd., Shanghai, China), super instrument (Shenzhen Fan Gao Wei Electronics Co., Ltd., Shenzhen, China), low-speed centrifuge (JW-1048, Anhui Jiawen Instrument Equipment Co., Ltd., Anhui, China), refrigerated centrifuge (centrifuge 5424, Eppendorf), electronic analytical balance (JA2003, Shanghai Jing Ke balance Co., Ltd., Shanghai, China), three-temperature three-control water bath pot (DK-8D, Jintan Instrument Co., Ltd., Jiangsu, China), blending elf (MIX-3000, Hangzhou Mio Instrument Co., Ltd., Hangzhou, China), PCR instrument (T100TM Thermal cyvler, BIO-RAD), continuous spectral labelling instrument (VERSAmax, Molecular Devices), horizontal electrophoresis apparatus (JY600HC, Beijing Junyi Oriental electrophoresis equipment Co., Ltd., Beijing, China), automatic gel imager (ChampGel 5000, Beijing Saizhi pioneering Technology Co., Ltd., Beijing, China).

### 2.3. Collection and Preservation of Plasma and Stool Sample

We collected the whole blood of the study group and the control group in the morning. The blood samples that were collected in the morning were collected while the subjects were fasting. The whole blood was placed in the heparin sodium anticoagulant tube, and the blood collection vessel was gently shaken so that the blood and heparin sodium in the tube became fully fused. Then, the blood collection vessel was placed in a centrifuge, centrifuged at 3000 r/min for 10 min. The supernatant was collected in a sterile 2 mL cryopreservation tube and stored in a refrigerator at −80 °C.

We collected feces from the subjects in the morning and stored them in the refrigerator at −80 °C.

### 2.4. Plasma Sample Pretreatment

The plasma samples were extracted, ultrasonographed, redissolved, centrifuged, and subjected to a series of operations, and the supernatant was collected. Then, 80 μL of supernatant of the centrifuged sample was removed and left to wait for detection. In addition, 10 μL of supernatant was taken from each sample and mixed into quality control samples for testing [10].

### 2.5. Metabolomics Test

In this study, LC-MS was used to detect the samples. First, the samples were separated using liquid chromatography. The A phase of the liquid chromatography was a water phase containing 25 mmol/L ammonium acetate and 25 mmol/L ammonia water, and the B phase was acetonitrile. Gradient elution: 0–0.5 min, 95% B; 0.5–7 min, 95%–65% B; 7–8 min, 65–40% B; 9–9.1 min, 40–95% B; 9.1–12 min, 95% B. The flow rate of the mobile phase was set at 0.5 mL/min, the temperature of the chromatographic column was set to 25 °C, and the temperature of the sample tray was set to 4 °C. The volume of the sample for the chromatography was 2 μL for both positive and negative ions [11].

High-resolution mass spectrometry was used in this study. High-resolution mass spectrometry data collection was carried out through the IDA (information correlation acquisition) mode. In the IDA mode, the data acquisition software (Analyst TF 1.7, AB Sciex) automatically selects ions and collects the second-level mass spectrum data according to the first-order mass spectrometry data and preset standards. Each cycle selects the 12 ions with the strongest intensity greater than 100 to carry out two-level mass spectrometry scanning. The energy of collision-induced dissociation was 30 eV, and the cycle time was 0.56 s. The parameters of the ion source are as follows: GS1:60 psi, GS2:30 psi, CUR: 35 psi, TEM: 600 °C, DP: 60 V, ISVF: 5000 V (Pos)/−4000(Neg).

### 2.6. Detection of Intestinal Flora

Extraction of genomic DNA from fecal samples was performed according to the instructions of the fecal sample genomic DNA extraction kit. The concentration and purity (OD value) of the extracted DNA were detected via enzyme-linked immunosorbent assay (ELISA). If the detection is qualified (OD value is between 1.6–2.0), the next operation is carried out. If the detection is not qualified, it must be re-extracted. The V3–V4 region of 16SrDNA of qualified DNA was detected to have been amplified by PCR; the bacterial primer was 338F:806R:5’-GGACTACHVGGGTWTCT AAT-3’, and the amplification procedure is shown in Table 1. The integrity was detected using 1.8% agarose gel electrophoresis (90 V, 40 min) and displayed with a UV Gel imaging system.

### 2.7. Library Construction and Sequencing

The PCR products were purified, quantified, and homogenized to form a sequencing library, and then the quality was detected. The qualified libraries were sequenced via high-throughput sequencing on the Illumina HiSeq 2500 platform.

### 2.8. Data Processing and Analysis

ProteoWizard software was used to transform the original mass spectrum into the mzXML format. To better analyze the data, we first preprocessed the original data, including filtering the deviation value of a single peak based on the coefficient of variation, preserving the peak area data of single group null values of less than 50% or all-group null values of less than 50%. One half of the minimum value was used to fill the missing value, and the internal standard was used to standardize and normalize the data. Then, all the metabolites were searched and sorted in the known database. After obtaining the optimized data, a series of multivariate variable identification and analysis was carried out under positive and negative ion modes. First, we used SIMCA software to perform logarithmic transformation and par formatting and then carried out principal component analysis (PCA). However, due to the influence of relevant variables, the difference was dispersed to more principal components, which is not conducive to subsequent analysis, so orthogonal projections to latent structures-discriminant analysis (OPLS-DA) were also needed. SIMCA software was used to perform logarithmic transformation and UV formatting of data. OPLS-DA analysis of the first principal component and replacement test were carried out to enhance the reliability of the analysis [12], and all data retain two decimal places. SPSS 17.0 statistical analysis software was used to analyze the data. The data were expressed as mean ± standard deviation, and the difference was compared using one-way ANOVA or chi-square test. *p* < 0.05 showed that the difference was statistically significant.

After sequencing, the original data needed to be preprocessed. The original data preprocessing included three steps: PE read splicing, tag filtering, and chimerism removal. After such processing, effective tags were obtained. OTU clustering analysis was carried out by using Usearch software to optimize the sequence with a similarity level of 97%. The OTU-representative sequences were compared with the microbial taxonomy database to obtain the representative species information. According to the results of OTU analysis, the community composition of each sample in each taxonomic level (phylum, class, order, family, genus, species) of the two groups was counted, and the species richness table and community structure diagram were drawn using QIIME software and R language tools. The Simpson, ACE, Shannon, Chao1 and indices were calculated using Mothur software. Then, a diversity analysis was carried out. The species richness and diversity of a single sample were studied, and the dilution curve and rank abundance curve were drawn. Principal coordinates analysis (PCoA) was carried out using QIIME software based on the Unweighted UniFrac to analyze β diversity and to compare the differences in species diversity, community composition, and structure between different groups of samples. LEfSe analysis was used to find species with statistical difference among different groups. SPSS 17.0 statistical analysis software was used to analyze the data. The data were expressed as mean ± standard deviation, and the difference was compared using one-way ANOVA or a chi-square test. *p* < 0.05 showed that the difference was statistically significant.

### 2.9. Screening and Identification of Metabolites

The screening criteria for metabolites are as follows:

1. The maximum change (fold change) > 2 indicates that the metabolite content in the two groups must be 2 times greater than that of the lowest group. The goal was to reduce bias.
2. When *p* < 0.05, the difference in metabolite content between the study group and the control population was statistically significant according to one-way ANOVA.
3. VIP > 1, indicating that the contribution of metabolites to the difference between groups was greater than 1.

Before the subsequent metabolic information integration analysis, it is necessary to identify the screened metabolites. First, HMDB and other databases were used to compare the information of the first-order ion fragments to determine the species. After that, the mass fragment in masslynx is used to match the information of interest obtained from secondary mass spectrometry. The matching degree of mass spectra at all levels is represented by numbers. If the mass spectrometry identification score is greater than or equal to 0.5, it can be considered that the identification of the substance is successful, and the identified metabolites can be regarded as differential metabolic markers. The matching conditions are as follows [13]: matching databases: Lipidblast, Metlin, Chemspider, HMDB; the mass error of the first order mass spectrum must be lower than 3 ppm, and the error of the two orders mass spectrometer fragment must be lower than 3 ppm.

### 2.10. Retrieval of Metabolic Pathways

Metabonomics analysis platform MetaboAnalyst (http://www.metaboanalyst.ca/faces/ModuleView.xhtmL, 26 September 2019) was used to analyze metabolic pathways. The detailed analysis process is as follows:

4. The successfully identified differential metabolic markers into were introduced into MetaboAnalyst.
5. The type of pathway database was human.
6. Fisher’s exact test was used for path selection.
7. The topological analysis method chose relative centrality.
8. MetaboAnalyst displays all the matched pathways in the form of metabolic maps by calculating the *p* value of the paths and the path influence value of the path topology analysis.

### 2.11. Statistical Analysis

SPSS 17.0 statistical software was used to analyze the data, and the data were expressed as $\bar{x}\pm s$ the mean plus or minus standard deviation. The one-way ANOVA or chi-square test was used to compare the differences. *p* < 0.05 indicated that the differences were statistically significant.

## 3. Results

### 3.1. General Information

A total of 15 coal workers with stage I pneumoconiosis were included in this study as the study group. All the patients were male with no complications, and their ages were (41.00 ± 5.61) years old. In the same period, 15 volunteers without any history of disease from the Dong Pang Coal Mine Hospital Physical Examination Center in Xing Tai were selected as the control group. All the volunteers in the control group were male, and their ages were (40 ± 5.74) years old.

### 3.2. Total Ion Chromatography

**Figure 1:**
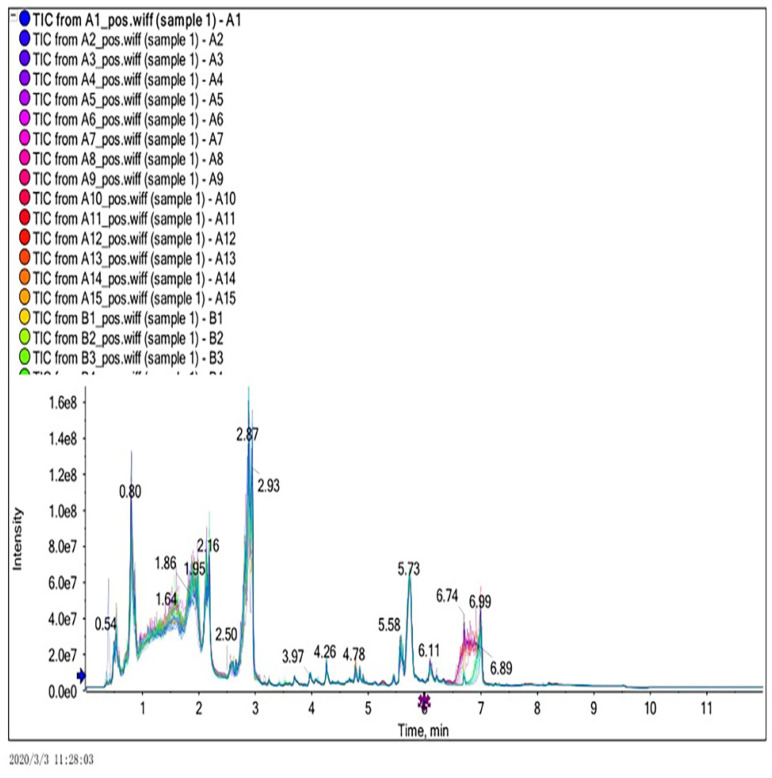
Total ion current diagram in positive ion mode.

**Figure 2:**
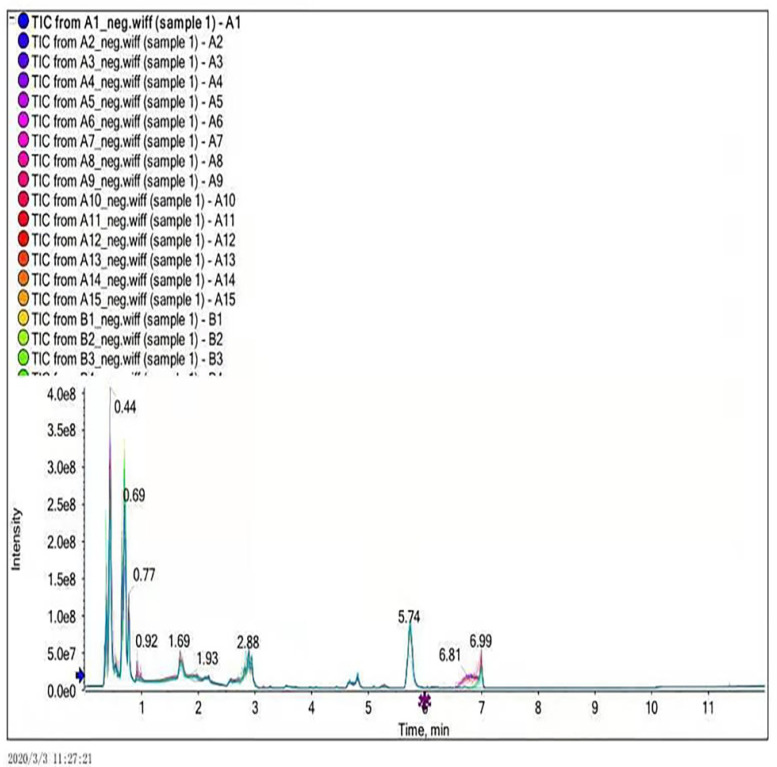
Total ion current diagram in negative ion mode.

In this experiment, ultra-performance liquid chromatography and ultra-time of flight mass spectrometry (UPLC/Q-TOF MS) were used to detect plasma metabonomics. The UPLC/Q-TOF MS system was used to collect metabolite information in plasma samples. Finally, 1534 type of ion characteristics under positive ion mode (Figure 1) and 1578 types of ions characteristics in negative ion mode (Figure 2) are obtained.

### 3.3. Quality Control Sample Analysis

**Figure 3:**
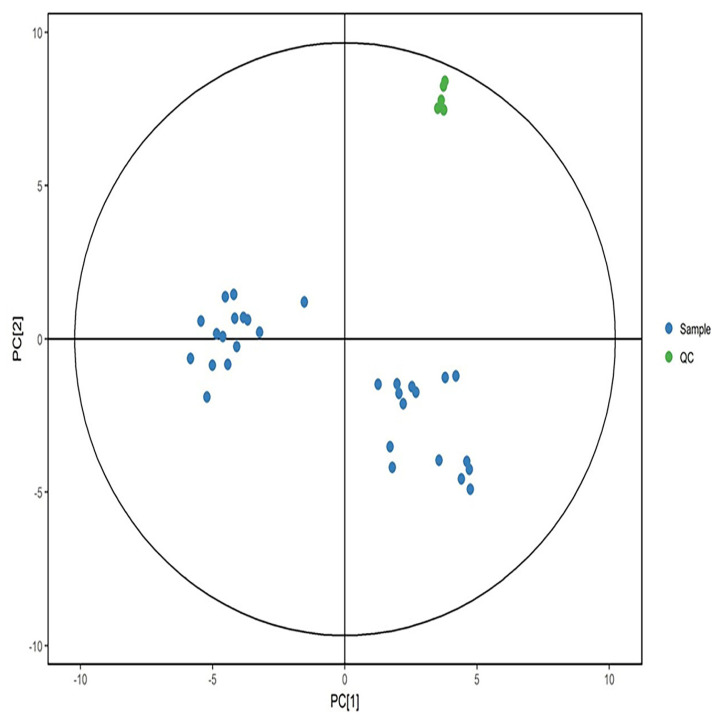
PCA score in positive ion mode. Green dots represent quality control samples, and blue dots represent study samples.

**Figure 4:**
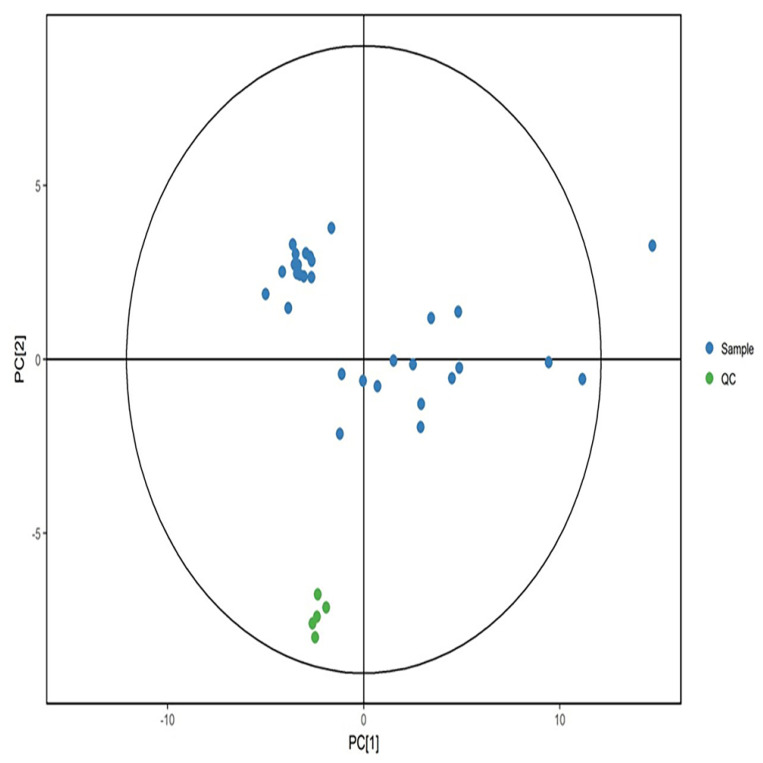
PCA score in negative ion mode. Green dots represent quality control samples, and blue dots represent study samples.

Quality control samples were aggregated in positive and negative modes and separated from the experimental samples significantly (Figure 3 and Figure 4). The results showed that the data quality is high, and the results are stable and reproducible.

### 3.4. PCA Score Map and OPLS−DA Score Map in Positive and Negative Ion Mode

**Figure 5:**
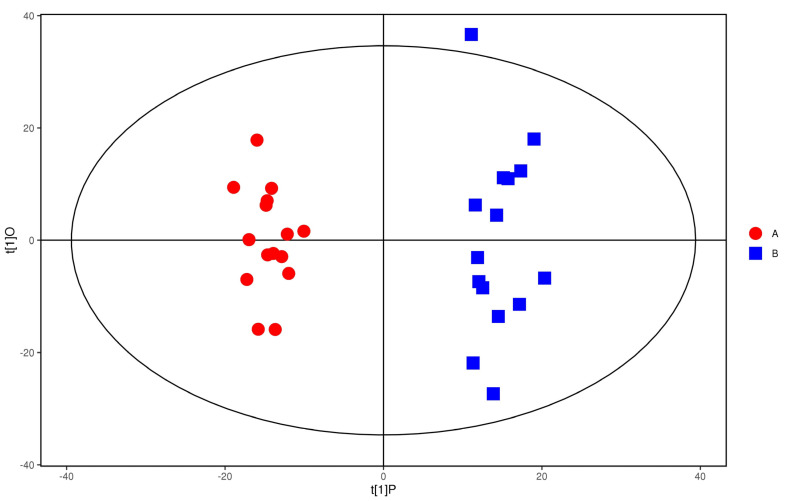
PCA score of positive ions. Red dots represent the control group, and blue dots represent the study group.

PCA scores of the plasma of the study group and control group in the positive ion mode are shown in Figure 5. As can be seen from the figure, the two groups of samples are completely separated, and there was no crossover phenomenon, which indicates that the plasma metabolic network of the study group and the control group has changed obviously.

**Figure 6:**
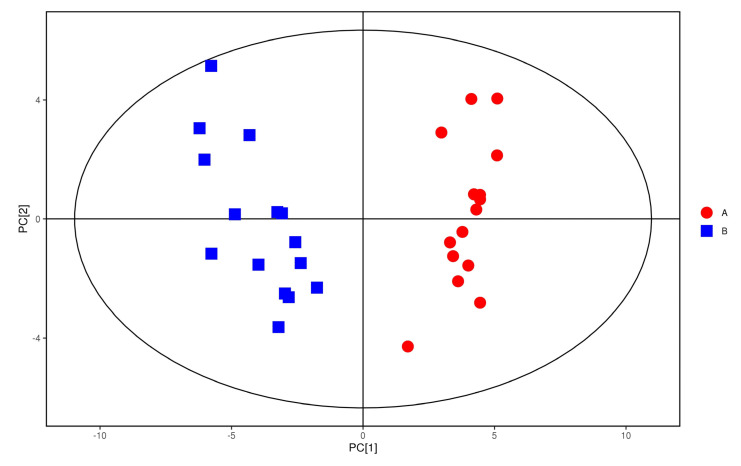
OPLS−DA score of positive ions. Red dots represent the control group. Blue dots represent the study group.

In order to pay more attention to the changes of plasma metabolites in coal workers’ pneumoconiosis, we must look for the coal workers’ pneumoconiosis-related metabolites. As shown in Figure 6, the distribution trend of plasma samples of the study group was significantly different from that of the control group in positive ion mode (R^2^X = 0.302, R^2^Y = 0.971, Q^2^ = 0.9). This indicated that there were significant differences in plasma metabolic phenotypes between study group and control group.

**Figure 7:**
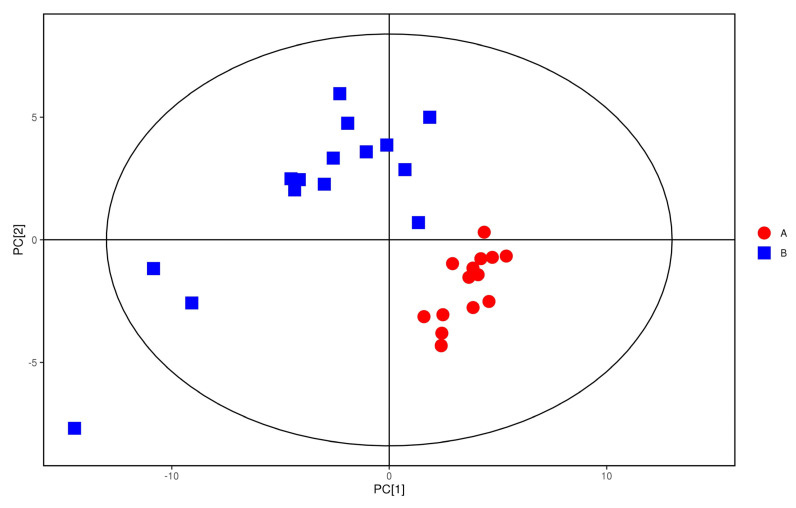
PCA score of anions. Red dots represent the control group. Blue dots represent the study group.

**Figure 8:**
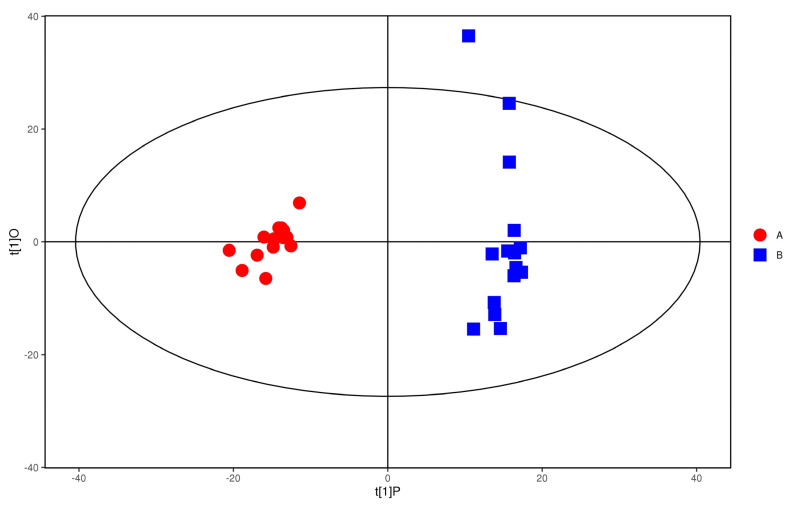
OPLS−DA score of anions. Red represent the control group. Blue represent the study group.

The plasma samples of patients in the study group were significantly separated from the plasma samples of the control group under the negative ion PCA score map, indicating that the plasma metabolic network of the study group and the control group changed significantly, as shown in Figure 7. The OPLS−DA model was used to illustrate the metabolic trend of plasma samples in the two groups. As shown in Figure 8, the plasma metabolic spectrum of patients with study group is completely separated from that of control group (R^2^X = 0.23, R^2^Y = 0.979, Q^2^ = 0.906). The result showed that the plasma metabolic phenotype of patients with study group was significantly different from that of the control group.

### 3.5. Permutation Test

**Figure 9:**
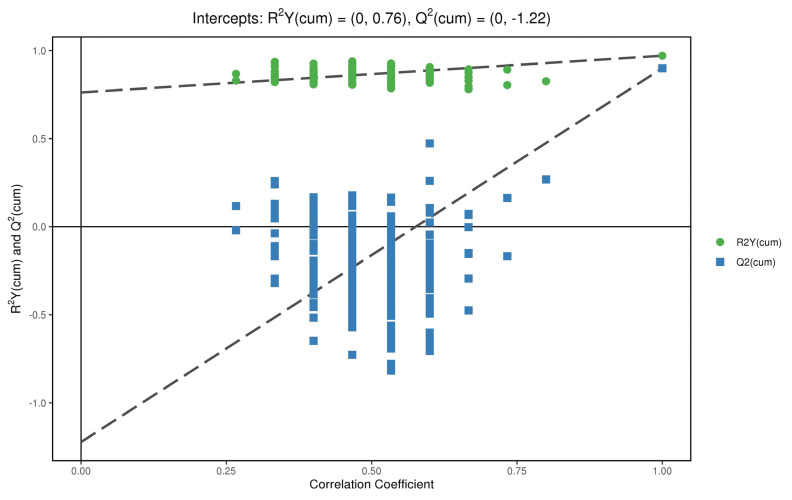
Displacement test chart of positive ions.

**Figure 10:**
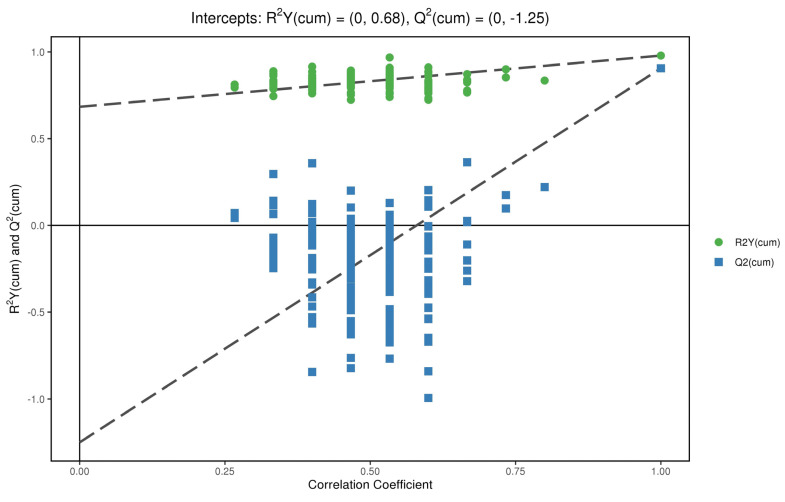
Replacement test of anions.

In order to test whether the OPLS−DA model is successful, we carried out a permutation test on the above OPLS−DA model (Figure 9 and Figure 10). R^2^Y is greater than 0.5 in the figure, which indicates that the model is successful. For the decrease in permutation retention, permutation variable Y was inversely proportional to the Q^2^ of the model. As can be seen from the figure, with the increase in Y, the increase of Q^2^ in decreases gradually, which indicates that the OPLS−DA model was successful, that the stability of the model was very good, and that there was no over-fitting phenomenon in the model.

### 3.6. Screening and Identification of Different Metabolites

**Figure 11:**
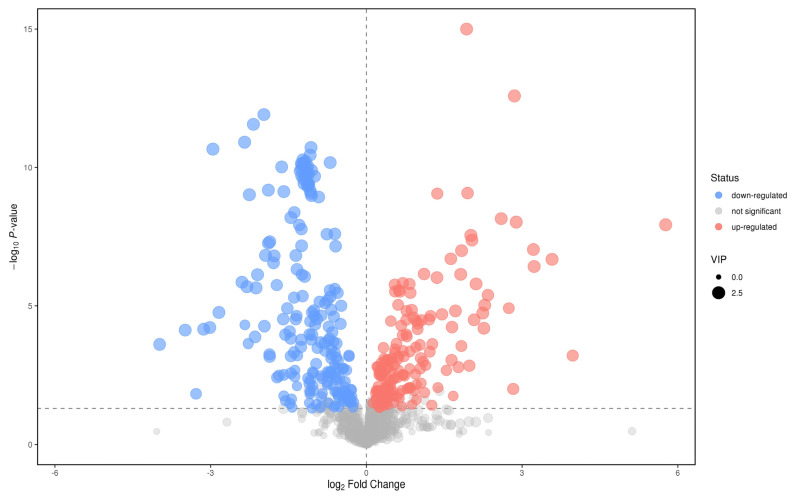
Volcanic diagram of positive ions in the plasma between study group and the control group. Abscissa was the change multiple of each substance in two groups. The ordinate is the *P* value of the *t* test, which is expressed by –logP. The dots in the picture represent metabolites, which represent the size of the VIP value. The red dots represent an increase in metabolites. The blue dots represent metabolites that are falling. Gray dots represent no difference in metabolic products.

In the positive ion mode, according to the *t* test *p* < 0.05, the first principal component of OPLS−DA model VIP > 1, and the maximum change multiple (fold change) > 2; significant differential metabolites were selected from the metabolites. A total of 49 different metabolites were screened (Figure 11). Through first− and second−level identification, it was determined that there were five kinds of differential metabolism: Diethanolamine, Leu−Leu, L−Glutamate, L−Tryptophan, and Phe−Trp. The contents of these five metabolites in the plasma of patients with CWP were higher than those of the control group (Table 2).

**Figure 12:**
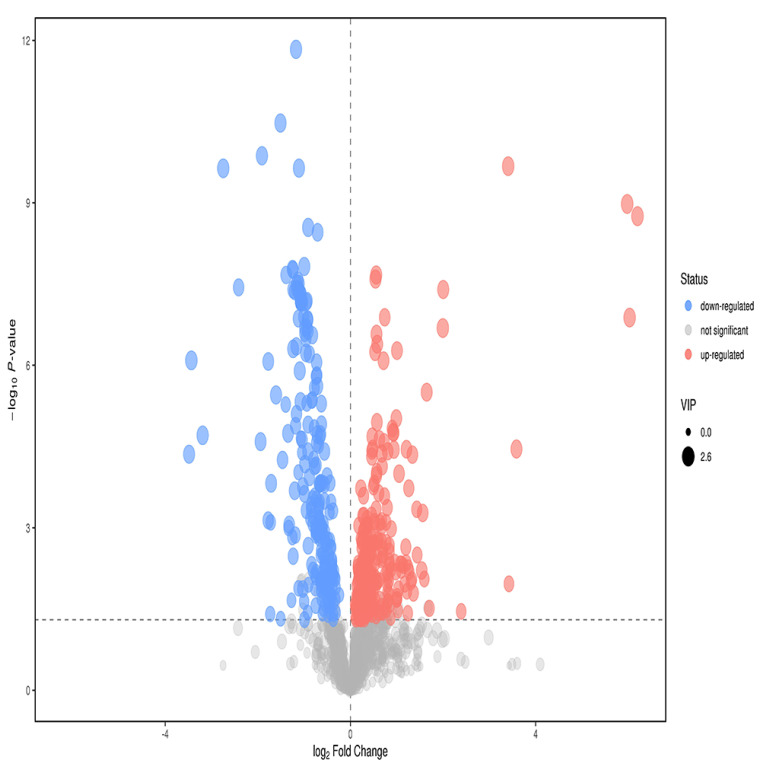
Volcano map of anions in the plasma of the study group and the control group. Abscissa is the change in the multiple of comparison between two groups of each substance, and ordinate is the *P* value of the *t* test, expressed by –logP. The point in the graph represents the metabolite, and the size of the point represents the size of the VIP value. Red dots represent ascending metabolites, blue dots represent descending metabolites, and gray dots represent undifferentiated metabolites.

T test *p* < 0.05; the VIP of the first principal component of OPLS−DA model > 1, and the fold change > 2. According to these two indices, the metabolites with significant differences were screened out from metabolites. Finally, 57 different metabolites were screened (Figure 12). Through primary and secondary identification, five different metabolites were identified as formylanthranilic acid, eicosapentaenoic acid, inosine, acamprosate and phenacemide. The contents of the above five metabolites in the plasma of study group were all higher than those of control group (Table 3).

### 3.7. Metabolic Pathway Analysis

Through the analysis of the metabolic map of the study group and control group in positive and negative ion modes, 10 metabolic markers related to the study group were selected and identified. These ten metabolites involved seven metabolic pathways. The seven metabolic pathways were glycerin phospholipid metabolism, D−glutamine and D−glutamic acid metabolism, cyanine metabolism, aminoacyl tRNA biosynthesis, nitrogen metabolism, tryptophan metabolism, and purine metabolism.

**Figure 13:**
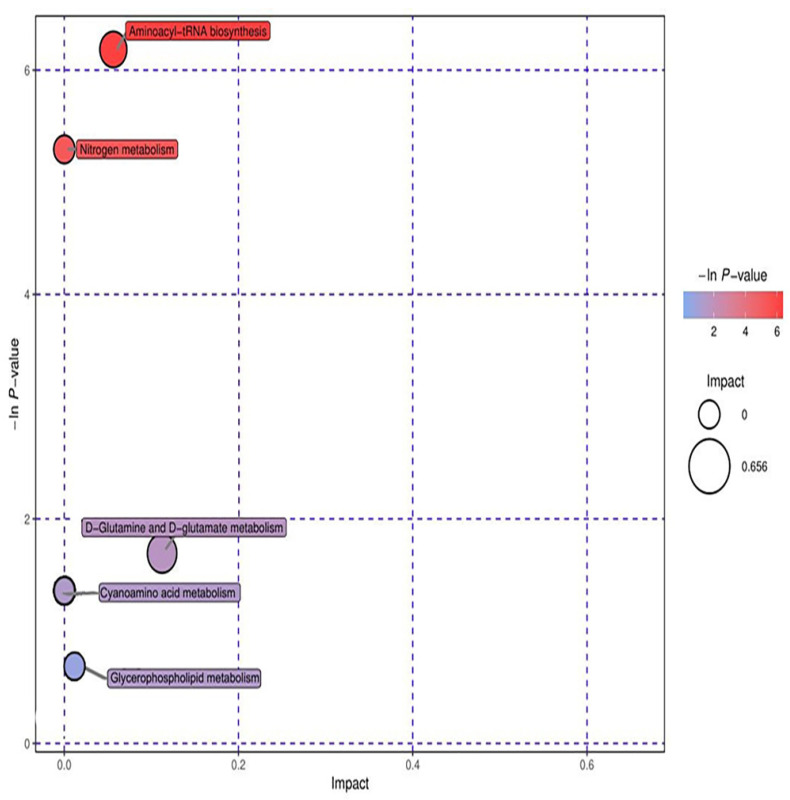
Topological analysis of positive ions. The ordinate of metabolic topological analysis was -ln(P), and the value of P represents significance. The lower the value of P and the higher the value of -lnP, the more significant the enrichment of metabolites in this pathway. The abscissa impact was the path influence value. The greater the influence value of the pathway, the more likely the metabolites of the query were consistent with the metabolites of the pathway.

**Figure 14:**
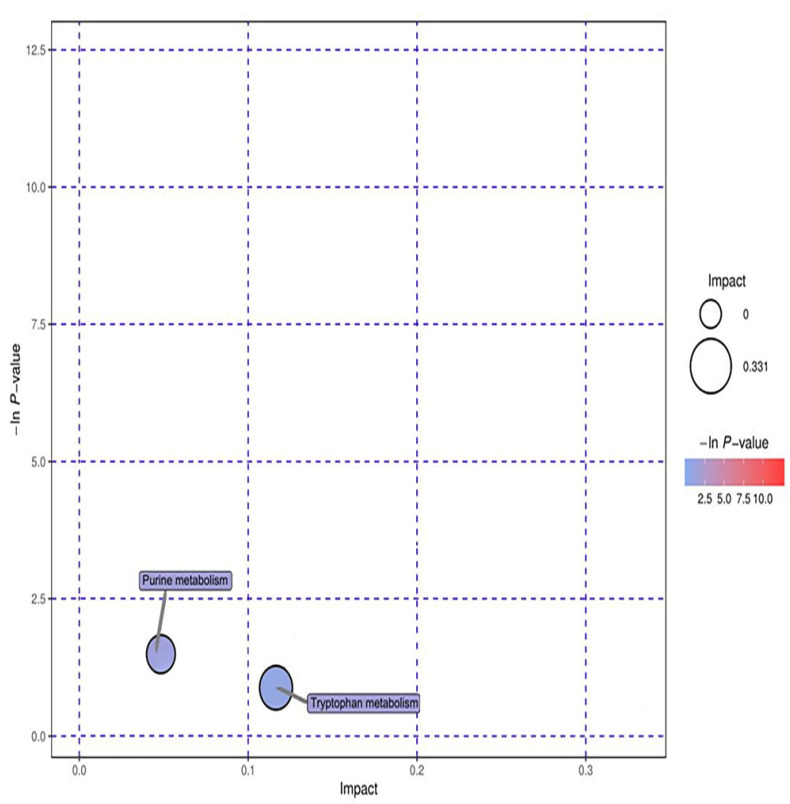
Topological analysis diagram of anion. The ordinate of metabolic topological analysis was -ln(P), and the value of P represents significance. The lower the value of P and the higher the value of -lnP, the more significant the enrichment of metabolites in this pathway. The abscissa impact was the path influence value. The greater the influence value of the pathway, the more likely the metabolites of the query were consistent with the metabolites of the pathway.

Ten significant metabolic markers were entered into MetaboAnalyst and then analyzed using metabolic topology (Figure 13 and Figure 14). The results showed that there was a metabolic pathway with obvious changes in the stage of the pneumoconiosis–aminoacyl tRNA biosynthesis pathway (*p* < 0.05).

### 3.8. Characterization of Differential Metabolites

Glutamic acid—chemical formula C_5_H_9_NO_4_, molecular weight 147.13—is an acidic amino acid. It is a colorless crystal which has a pleasant taste, is slightly soluble in water, and is soluble in hydrochloric acid. Glutamate is abundant in cereal protein and animal brains. Glutamate plays an important role in the metabolism of protein and participates in many important chemical reactions in animals, plants, and microorganisms.

Diethanolamine, abbreviated as DEA in English, is a colorless liquid or crystal. It is alkaline and can absorb gases, such as carbon dioxide and hydrogen sulfide, in the air. Cough, headache, nausea, vomiting, and coma can occur after high-concentration inhalation. Prolonged skin contact may lead to burns. Chronic effects include nausea, vomiting, and abdominal pain after massive oral administration. Long-term contact may cause liver and kidney damage.

Tryptophan, chemical formula C_11_H_12_N_2_O_2_, is one of the essential amino acids of the human body. It is a white or light yellow crystal or crystalline powder that is odorless and slightly bitter. It is slightly soluble in water, easily soluble in formic acid, and soluble in sodium hydroxide solution or diluted hydrochloric acid. Tryptophan is an important precursor of auxin biosynthesis, which widely exists in higher plants. It is also a precursor of serotonin, which is an important neurotransmitter in the human body.

Eicosapentaenoic acid is an organic compound with the molecular formula C_20_H_30_O_2_ and a molecular weight of 302.451. At room temperature, it is a colorless to pale yellow transparent liquid. It is tasteless and odorless and has a unique smell after oxidation. EPA is obtained by eating cold-water fish, such as wild salmon (not raised in fishing grounds), mackerel, sardines, and herring.

Inosine—also known as hypoxanthin and hypoxanthine nucleoside, with the chemical formula C_10_H_12_N_4_O_5_—is a nucleoside compound formed by the combination of hypoxanthine and ribose. In de novo synthesis of purine, inosinic acid can be used as a precursor for the synthesis of adenylate and guanosine acid. It can be used for treating leukopenia, thrombocytopenia, various heart diseases, acute and chronic hepatitis, liver cirrhosis, etc., resulting from various causes. In addition, it can also be used to treat central retinitis, optic nerve atrophy, etc.

Acacia acid, also known as acetyl taurine, is called acamprosate in English. It has the molecular formula C_5_H_11_NO_4_S, a molecular weight of 181.2101, and a density of 1.336 g/cm^3^. It can be used to treat alcohol addiction, act on the reward system of brain, and block the enhancement mediated by part of alcohol γ-aminobutyric acid (GABA).

**Figure 15:**
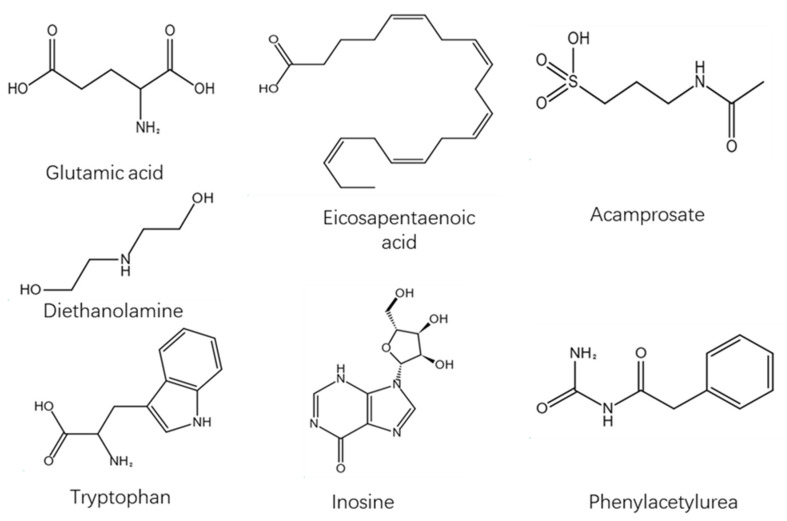
Structural formula of differential metabolites.

Phenylacetylurea, the molecular formula is C_9_H_10_N_2_O_2_. This product is an organic synthetic intermediate, which is used for the synthesis of anti-tuberculous drugs, aminothiourea and sulfonamide drugs, as well as rodenticides. It can also be used as an analytical reagent for the quantification of chromium and the identification of aldehydes and ketones. In addition, the product is also used as an intermediate of pesticide, rubber additives, and synthetic resin additives (Table 4, Figure 15).

### 3.9. SrDNA Intestinal Flora Detection Results

#### 3.9.1. Sequencing and Sequence Length Distribution of Bacteria

Based on Illumina HiSeq 2500 platform, the DNA fragments from intestinal flora were sequenced at two ends. A total of 2,154,846 pairs of readings were obtained from 30 samples. A total of 1,894,561 clean tags were formed by read splicing and filtering at both ends. At least 74,239 clean labels were generated for each sample, and 75,642 clean labels were generated on average. The average sequence length was mainly distributed between 400–430 bp (Table 5).

#### 3.9.2. OTU Classification

**Figure 16:**
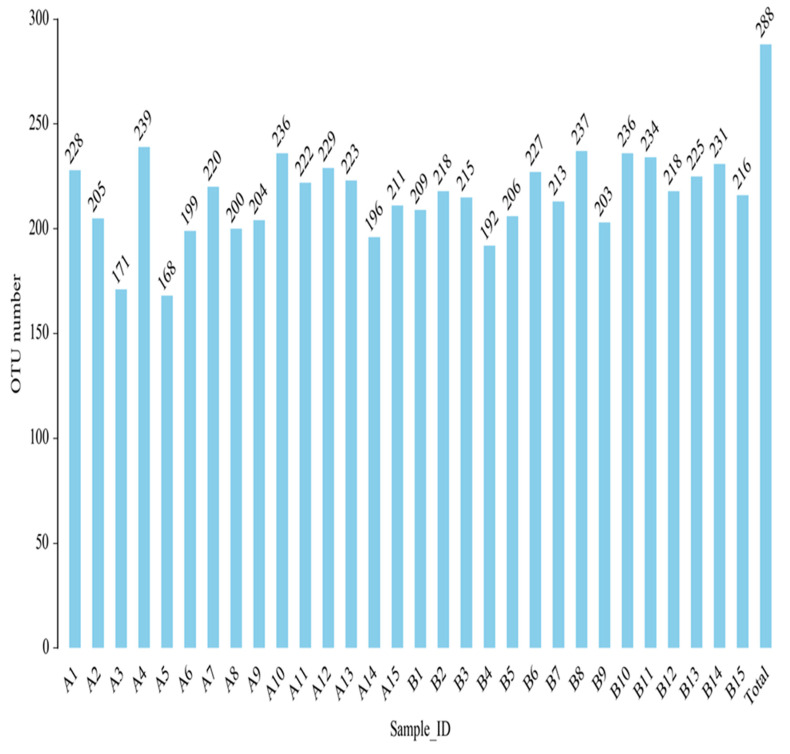
Histogram of OTU quantity of fecal intestinal flora of each sample after clustering.

OTU (operational taxonomic unit) is an artificial taxonomic unit with similar species and genera. According to the criteria, 97% sequence similarity is more than or equal to the same OTU—that is, the same microbial species. The number of OTU in each sample group was obtained (Figure 16).

**Figure 17:**
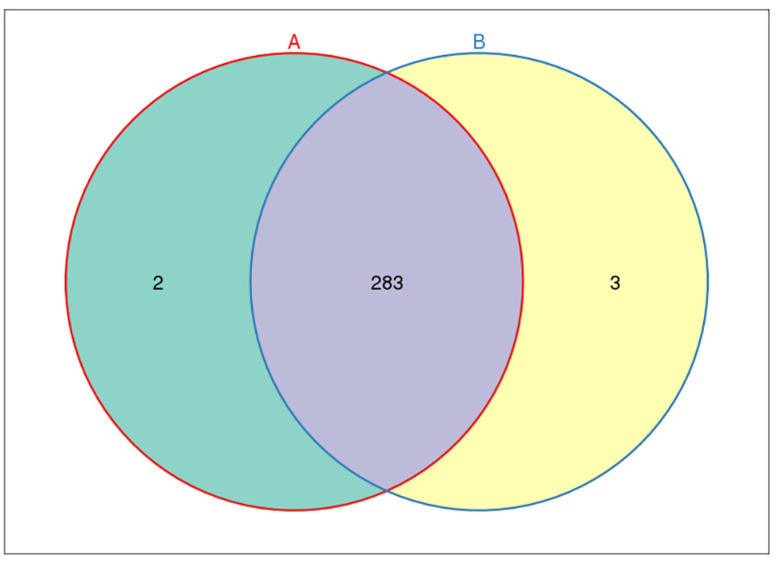
Common and unique OTUs of fecal intestinal flora among groups. Each circle represents the number of fecal microbial OTUs in the feces of each group. The intersection part of the circle represents the number of OTUs shared between groups. The non-intersecting parts represent the number of exclusive OTUs in each group.

From Venn diagram, we could clearly see the number of common and unique OTUs among groups, and the situation of OTU between groups. A total of 285 OTUs were identified in fecal microorganisms in the control group, while 286 OTUs were found in the study group. There were 283 OTUs of fecal microorganisms in patients in the study group and control group. There were three unique OTUs in patients in the study group and two unique OTUs in the control group (Figure 17).

#### 3.9.3. Species Annotation and Cluster Analysis

**Figure 18:**
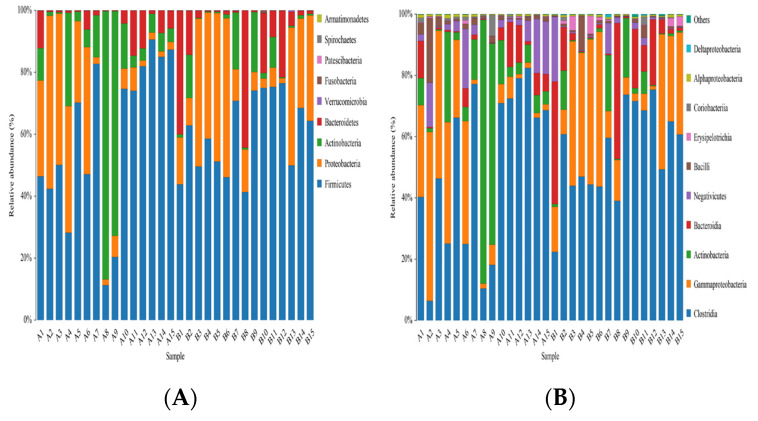

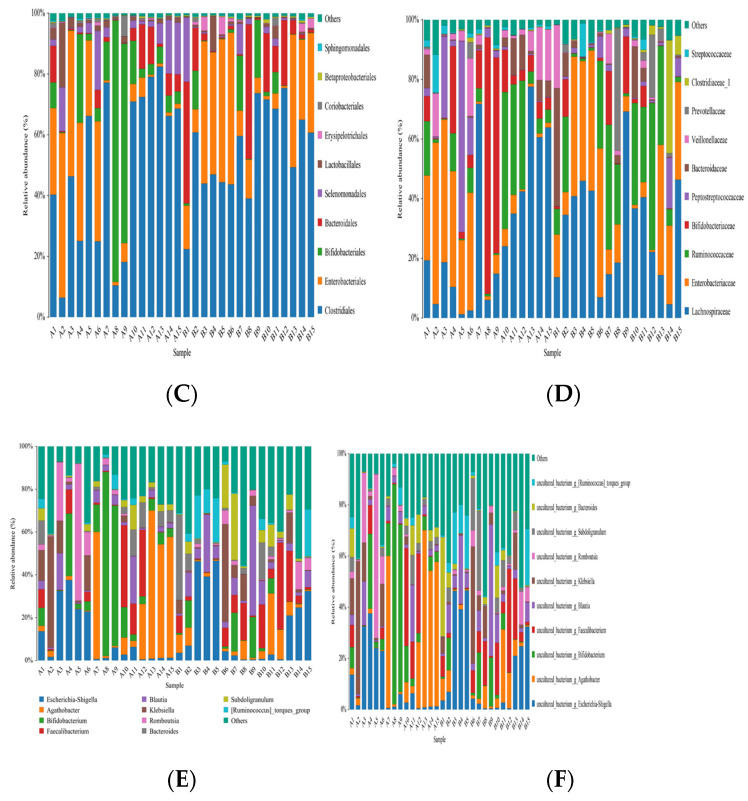
The histogram of species distribution of each sample at the levels of phylum, class, order, family, genus, and species in Figures (**A**–**F**).

The representative sequences of OTUs were compared with a microbial database to obtain the species information of each OTU. Then, biological classification was carried out to describe the community composition of each sample. The results of OTU classification showed that the number of OTUs of fecal microorganisms in patients in the study group was higher than that of the control group at the classification levels of phylum, class, order, family, genus, and species (*p* < 0.05). There was no difference in order and family level (*p* > 0.05) (Table 6). At the same time, two groups of species were displayed in the form of histogram in the distribution level of the phylum, class, order, family, genus, and species (Figure 18).

#### 3.9.4. Analysis of Alpha Diversity

Alpha diversity could show the number and diversity of species in each sample. There are many indicators showing the number of species. Simpson, Chao1, Ace, and Shannon could be used to represent the species diversity index. For the same number of species, the greater the evenness of each species in the community, the greater the diversity of the community. The Shannon index was inversely proportional to the Simpson index. If the Shannon index was large, it means that the Simpson index was small, which meant that there are many species in the sample. In this experiment, the statistics of α diversity index values of samples are as follows (Table 7). Compared with control group, there was no significant difference in the Ace, Chao1, Shannon, and Simpson indices of the study group (*p* > 0.05).

**Figure 19:**
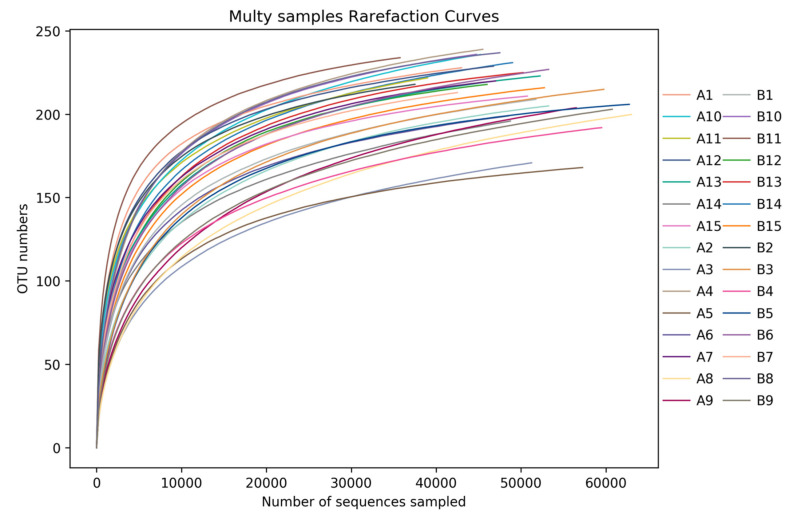
Rarefaction curve of each sample fosr each group. Abscissa was the number of sequences, and ordinates represented the number of OTU in sequence clustering. The curves of different colors represented various samples.

1. Dilution curve;

From the dilution curve of each sample (Figure 19): the curve gradually levelled down as the sequencing volume reached 20,000, indicating that the number of species did not increase significantly with the increase of sequencing times in such an environment. The sequencing quantity of these 30 samples was more than 20,000, which indicated that the sequencing depth met the requirements of comprehensive data analysis and could reflect the status of bacterial flora.

**Figure 20:**
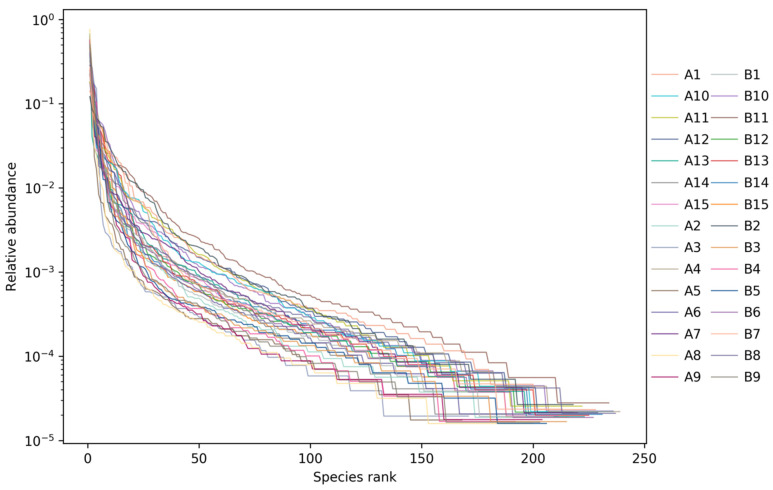
Rank abundance curve of each sample for each group. The abscissa represented the OTU level, and the ordinate represented the relative abundance. Each sample was marked with different color curves.

2. Rank abundance curve;

The rank abundance curve of each sample (Figure 20) showed that when the number of OTUs reached 150, the curve tended to be gentle, representing a uniform distribution of species.

#### 3.9.5. Analysis of Diversity in Bacteria

**Figure 21:**
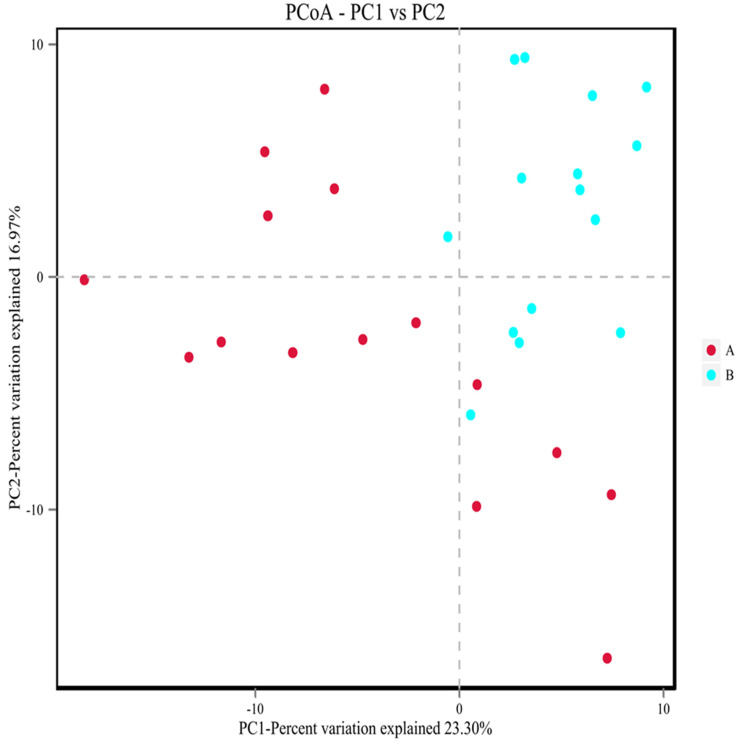
PCoA analysis of microbial communities in each group. A showed the control population group. B showed the study group. Points in the figure represent each sample separately, and groups are distinguished by different colors. The transverse and ordinate coordinates are the two eigenvalues (%) that cause the greatest difference between the samples and represent the main degree of influence.

Beta diversity analysis could reflect the similarity of species diversity among different samples by comparing the distances between different samples. The closer the distance, the higher the similarity of flora. Its analysis algorithm mainly includes two categories: weighted and unweighted. The difference between the two groups was whether the richness of species is considered. Because of the complex diversity of micro-organisms and the huge differences of species composition among different groups, unweighted methods are more commonly used. In this study, the PCoA analysis of unweighted UniFrac distance was used to understand the difference among bacteria. As shown in Figure 21, under the principal coordinate PC1 = 23.30%, the study group and the control group are not completely separated into approximately two microbial communities that do not interfere with each other, but there was a separation trend. This indicated that there are differences in intestinal flora between the study group and the control population.

#### 3.9.6. LEfSe Analysis of Samples between Groups

**Figure 22:**
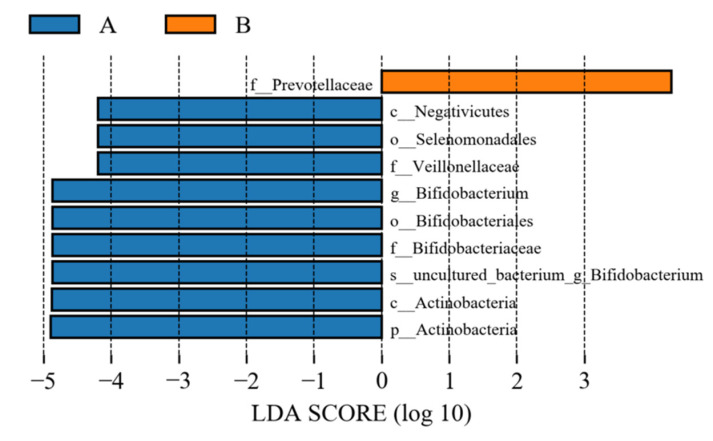
Histogram of LDA value of intestinal flora. A represents the control group; B represents the study group.

**Figure 23:**
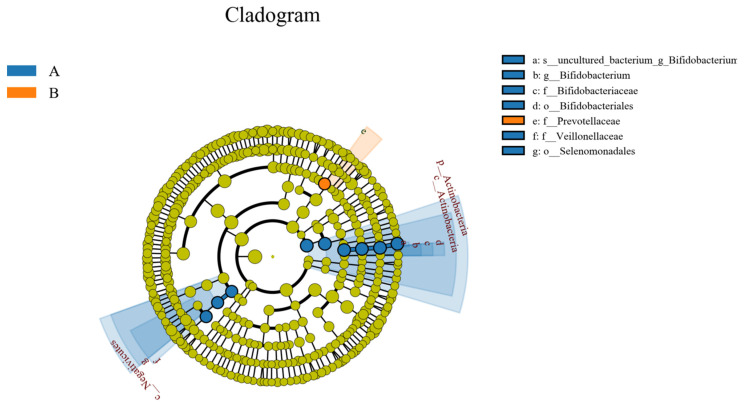
Evolutionary branch map of intestinal flora LefSe. A represents the control group; B represents the study group.

LEfSe (line discriminant analysis (LDA) effect size) analysis could detect the number of flora in the two groups and could also detect the species with statistical significance. LEfSe analysis included LEfSe analysis of an evolutionary branching diagram and LDA value distribution histogram. In this study, the LDA value was set to 4.0. As shown in Figure 22 and Figure 23, compared to the control group, the abundance of the intestinal flora Veillonellaceae, Negativicutes, Actinobacteria, Bifidobacteriales, Selenomonadales, Actinobacteria, Bifidobacteriales-uncultured, Bifidobacteriaceae, and Bifidobacterium decreased in the study group, and that of Prevotellaceae increased.

#### 3.9.7. Characterization of Differential Flora

Veillonellaceae is Gram-negative anaerobic cocci. The cell diameter was 0.3–2.6 μm. Bacteria are basically arranged in pairs, but also appear singly or in a chain. The chain is connected by pairs of cocci, and the adjacent side of pairs of cocci can be flat with no spores, no movement, and no flagella. Although the Gram stain is negative, it tended to remain purple. Fermentation of organic matter produces acid and gas, some of which are non-fermented carbohydrates. Veillonellaceae is parasitic in the intestines of warm-blooded animals, such as humans, ruminants, rodents, and pigs.

Actinomycetes are a special type of prokaryote which can form branched hyphae and conidia. They grow in the form of hyphae and mainly reproduce through spores. They are so named because of their radial groups. Most of them have well-developed branch hyphae. The hypha is thin, and its width is close to that of rod-shaped bacteria—approximately 0.2–1.2 μm. They can be divided into vegetative hyphae, also known as basal hyphae or primary hyphae. The main function of vegetative hyphae is to absorb nutrients, and some can produce different pigments, which is an important basis for strain identification. Aerial hyphae, superimposed on vegetative hyphae, are also known as secondary hyphae. When aerial hyphae develop to a certain stage, they can differentiate into spores to form hyphae.

Bifidobacterium is a Gram-positive, inactive, rod-shaped, sometimes bifurcated, and strictly anaerobic bacterium. It exists widely in the digestive tract, vagina, oral cavity, and other habitats in humans and animals. Bifidobacteria is an important member of the intestinal flora of humans and animals. Some strains of Bifidobacterium can be used as probiotics in food, medicine, and feeds.

Prevotellaceae is a polymorphous bacterium which does not produce spores, does not move, and is strictly anaerobic. It can metabolize organic nutrients and has a moderate ability to break down sugar. When grown in glucose broth, it easily formed smooth or linear precipitation, and the final pH value reached 4.5. The utilization rate of glucose was 30–39%. The main fermentation products were acetic acid, succinic acid, and small amounts of isobutyric acid, isovaleric acid, and lactic acid (Table 8 and Table 9).

#### 3.9.8. Relationship between Human Fecal Flora and Plasma Metabonomics

**Figure 24:**
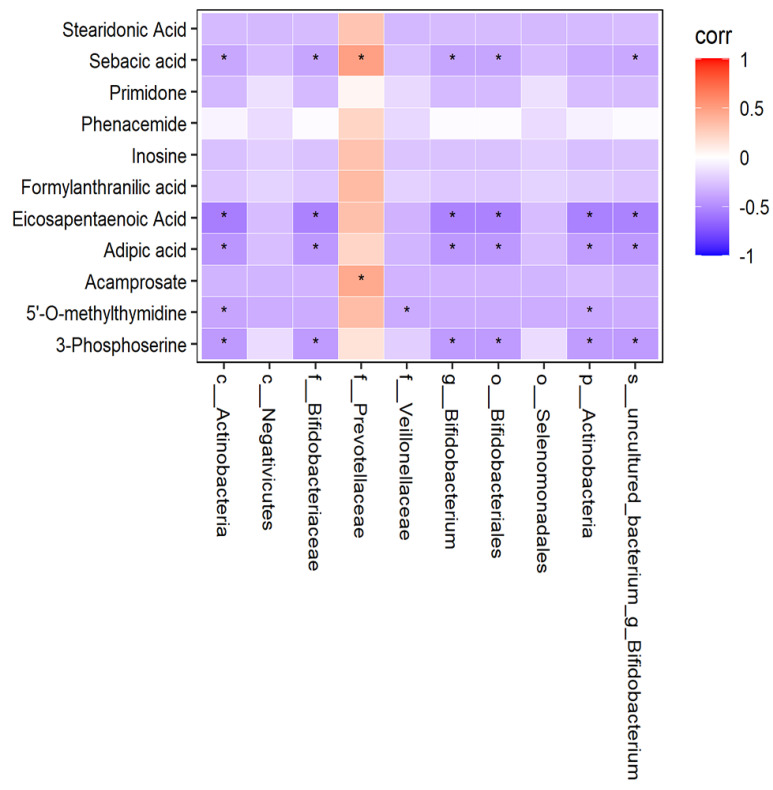
Correlation thermodynamic diagram in negative ion mode.

**Figure 25:**
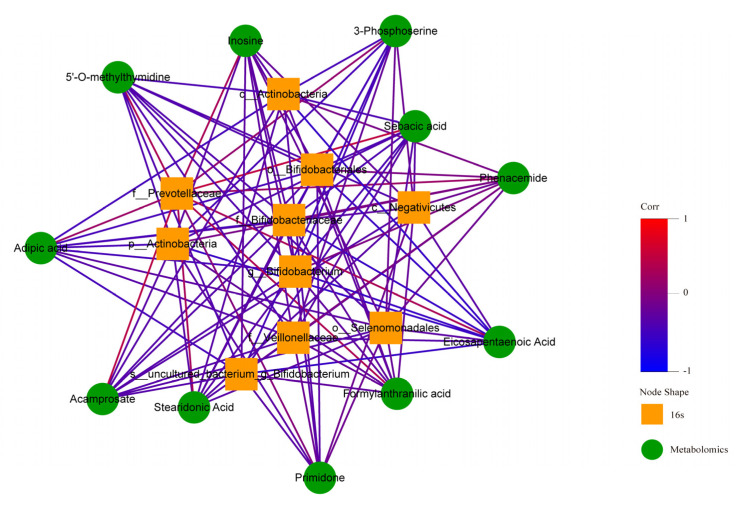
Correlation network diagram in negative ion mode.

In order to find out whether there was a correlation between human fecal flora and human plasma metabonomics, we have conducted correlation analysis and correlation network analysis. The thermodynamic diagram and correlation network diagram (Figure 24 and Figure 25) show that eicosapentaenoic acid was negatively correlated with c_Actinobacteria, p_Actinobacteria, Bifidobacterium_uncultured, Bifidobacteriaceae, Bifidobacterium, and Bifidobacteriales in the negative ion mode. Acamprosate was positively correlated with prevotellaceae. The above results were statistically significant. In addition, formylanthranic acid, inosine, and phenacemide were also related to the flora, but the results are not statistically significant. Sebacia acid, adipic acid, and other metabolites were also correlated with the flora, and the results are statistically significant. However, these metabolites are not included in the differential metabolites we screened.

**Figure 26:**
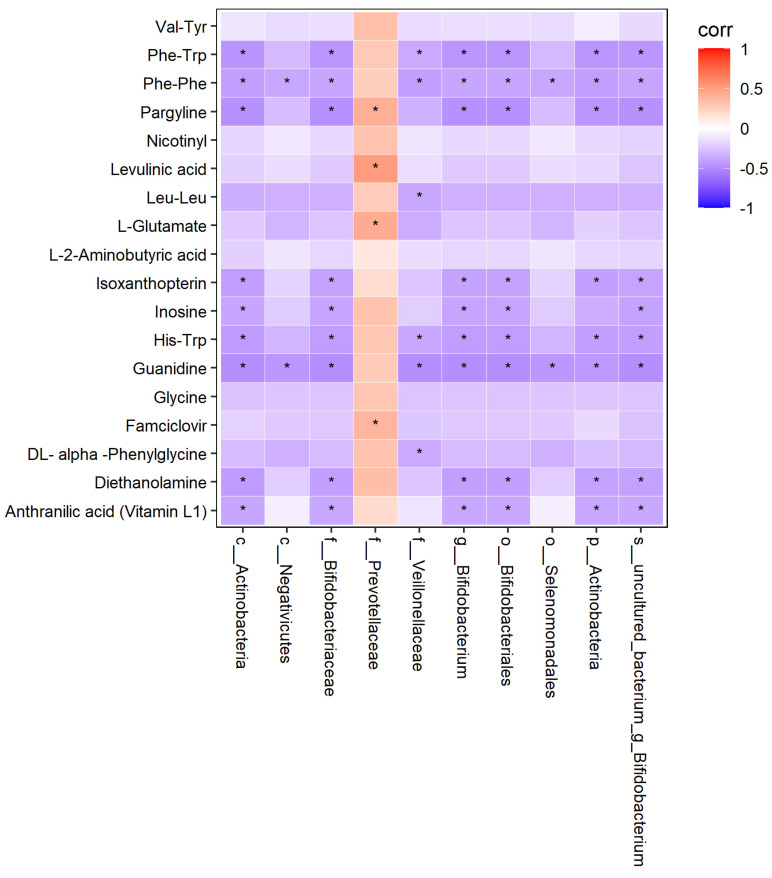
Correlation thermodynamic diagram in positive ion mode. In the figure, red (corr = 1), blue (corr = −1), white (corr = 0). The data with correlation P value less than 0.05 were marked with “*” in the figure. The ordinate was metabonomics differential metabolites, and the abscissa was 16S differential flora.

**Figure 27:**
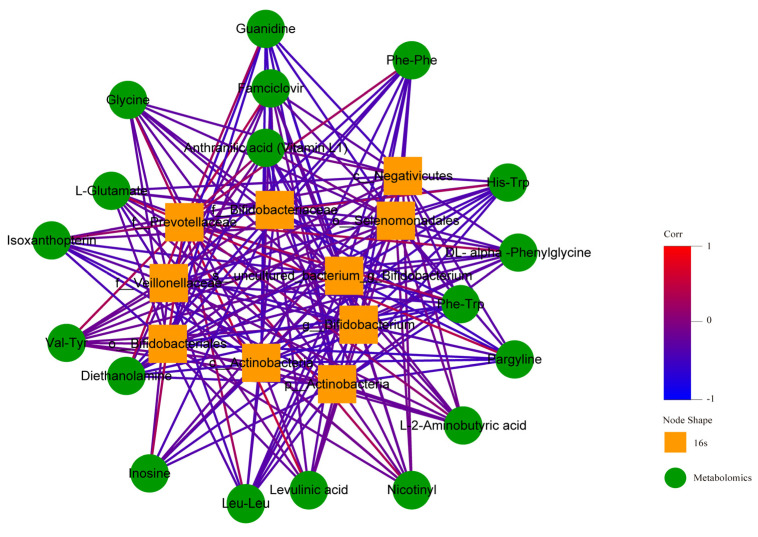
Correlation network diagram in positive ion mode. In the figure, the green circle represents the differential metabolites. The yellow square represents the differential flora. The line represents the correlation coefficient between the differential metabolites and the differential flora. The red line represents the positive correlation, and the blue line represents the negative correlation.

The thermodynamic diagram and correlation network diagram (Figure 26 and Figure 27) show that Phe-Trp was negatively correlated with c_Actinobacteria, p_Actinobacteria, Bifidobacterium_uncultured, Bifidobacteriaceae, Bifidobacteria, Bifidobacterium, and Veillonellaceae in the positive ion mode. There was a negative correlation between Leu-Leu and Veillonellaceae. There was a positive correlation between L-Glutamate and Prevotellaceae. Inosinee was negatively correlated with c_Actinobacteria, p_Actinobacteria, Bifidobacterium_uncultured, Bifidobacteriaceae, Bifidobacteria, and Bifidobacterium. Diethanolamine was the same as inosine. The ten different metabolites selected included the above five metabolites, and the above results have statistical significance. The remaining 13 metabolites were related to the flora, but the results of 9 metabolites were statistically significant, while those of 4 metabolites were not statistically significant.

## 4. Discussion

It is reported that pneumoconiosis threatens the health of hundreds of millions of workers exposed to dust all over the world, and its prevalence and incidence in developing countries are very high. At present, the pathogenesis of pneumoconiosis was not clear, and there is no effective treatment. At present, some studies have carried out metabonomic analysis of COPD, idiopathic pulmonary fibrosis (IPF), and asthma and summarized some different biomarkers [5]. However, this is different from the metabolic difference between the study group and the control group found in this study. In this study, based on the UPLC-Q/TOF-MS analysis method, we analyzed and compared the plasma metabolic profiles of the study group and the control group. In addition, 10 endogenous metabolites related to pneumoconiosis were identified by multivariate and univariate statistical analysis methods. The 10 metabolites were formylanthranilic acid, Leu-Leu, inosine, eicosapentaenoic acid, acamprosate, phenacemide, diethanolamine, L-glutamate, L-tryptophan and Phe-Trp. Further topological analysis of the related metabolic pathways involved in these metabolic markers revealed a metabolic pathway, the aminoacyl-tRNA biogenesis metabolic pathway, with obvious changes in pneumoconiosis stage. Therefore, we will focus on the metabolic pathway of the aminoacyl tRNA biosynthesis pathway, which was more obvious in the metabolic pathway analysis.

In this study, under the positive and negative ion mode, we all screened out a metabolite–tryptophan. Tryptophan, an essential amino acid in human body, is an aromatic amino acid with relatively low content in plasma and body fluid [14]. There were two metabolic pathways for tryptophan; one is that indoleamine-2,3-dioxygenase (IDO) catalyzes the degradation of tryptophan in the tyrosine ammonia pathway, and the other depends on the tetrahydro pyrimidine tryptophan pathway [15]. Tryptophan not only synthesizes protein in vivo, but its metabolites also play an important role in regulating the mood and metabolism of the human body. Tryptophan is mainly involved in humoral immunity. Moreover, a large amount of research evidence shows that the immunity of the body is closely related to the disorder of tryptophan metabolism. Studies found that tryptophan metabolites affected the expression of immune cells, and thus affect the immunity of T cells [16]. At the same time, studies showed that the occurrence of pneumoconiosis was the result of immune dysfunction and a variety of cytokines [17,18,19]. In this study, it was found that the content of tryptophan in the plasma of study group was significantly increased. In addition, tryptophan tRNA synthase (TTS) can accelerate the combination of tryptophan and tRNA Trp, increase the reserve of tryptophane-tRNA needed for protein synthesis in cells, and counteract the immunosuppression caused by IDO [20] so as to keep the body in a balanced state. Tryptophan is an essential substance for in the synthesis of tryptophan tRNA synthetase (TTS). Therefore, in this study, we further analyzed the topology of related metabolic pathways involved in metabolic markers and found an aminoacyl-tRNA biosynthesis metabolic pathway with significant changes in the stage of pneumoconiosis. Therefore, this study speculated that the increase in tryptophan in plasma may be due to the disorder of the synthesis pathway of tryptophanyl-tRNA, resulting in the decrease in synthesized tryptophanyl-tRNA synthetase and the subsequent increase in tryptophan concentration. It was mentioned in the above article that the body immunity of pneumoconiosis patients was decreased, while the plasma tryptophan concentration of pneumoconiosis patients was increased. Therefore, the body immunity of pneumoconiosis patients was probably decreased because the disorder of the aminoacyl-tRNA synthetic pathway in the body caused the reduction of synthetic chromoyl-tRNA synthetase, which weakened the ability to fight against IDO-mediated immunosuppression and thus led to the body immunity decrease of pneumoconiosis patients. This must be confirmed by further studies.

Some studies have found that the metabolism of the body will be affected by the metabolism of intestinal flora. Intestinal flora can make use of dietary fiber in food, produce monosaccharide, and lactic acid and can also form short-chain fatty acids [21]. Short-chain fatty acids formed by intestinal flora are important energy sources for hosts and microorganisms. In addition, intestinal bacteria can also metabolize branched chain amino acids to branched chain fatty acids. In addition, the synthesis of vitamins in the human body is closely related to the metabolism of intestinal flora. Intestinal flora plays an important role in maintaining human health. Bifidobacterium and Lactobacillus belong to probiotics, which are the dominant intestinal flora. They have the functions of promoting digestion, producing multiple vitamins, enhancing immunity, eliminating endotoxin, anti-tumor infection, etc. Escherichia coli, Enterococcus faecium, and Enterococcus faecalis belong to opportunistic pathogens, also known as intestinal non-dominant flora. When they proliferate in large quantities, they can enhance their invasive ability and cause damage to intestinal mucosa, thus causing a series of inflammatory reactions [22,23]. Under physiological conditions, various intestinal flora—as well as intestinal flora and the outside world together—form a stable state, which is called intestinal flora microecology and plays an important role in maintaining the steady state of the human machine environment. For example, it has the functions of substance metabolism, providing a natural biological barrier, promoting proliferation and differentiation of intestinal epithelium, and regulating immune function [24]. Cell lysis with a molecular weight of 700,000 can produce endotoxin, which releases lipopolysaccharide after the cell walls of Gram-negative bacteria dissolve [4]. Intestinal bacteria are the most abundant bacteria in the human body, and most of the body’s endotoxin comes from Gram-negative bacteria in the intestinal tract. Some studies have pointed out that the morbid state of the large intestine will lead to the rapid propagation of bacteria in the intestinal tract, which will be absorbed into the blood with endotoxin, destroying the shielding function of the intestinal tract. Intestinal endotoxin reaches the lungs through the blood, causing lung diseases. Endotoxemia, in turn, leads to disorder of gastrointestinal function, decrease in muscle tension, and weakening of intestinal peristalsis. Intestinal dilatation increases the permeability of the capillary wall, which causes a large amount of inflammatory substance to seep out. The metabolism of pneumoconiosis patients shifts through intestinal mucosal barrier, thus causing a vicious cycle of enteropathy and lung disease. Other studies have shown that the gut is the target organ of metadata object description schema (MODS), and it is also an essential factor in the initiation of MODS [25].

The intestinal flora of the study group and the control group were subjected to high-throughput sequencing, and the results showed that the sequencing depth was sufficient to meet the requirements of the experiment. Previous literature reported that environmental dust exposure (such as to arsenic, cadmium, lead, and other heavy metals) would lead to the disorder of intestinal flora and suggested that the disorder of intestinal flora might be one of the mechanisms by which heavy metal exposure induced some diseases and damaged the health of the population [26]. Some researchers found that the intestinal flora of silicosis patients was imbalanced and that the abundance of some flora species was different by comparing silicosis patients with control group [27]. In this study, α diversity and β diversity were used to reflect species diversity. The study found that the intestinal microbial communities in the two groups were separated from each other and that their species diversities were also different. After correlation analysis, it was found that there was a correlation between different metabolites and intestinal flora. It was found that the intestinal microbial communities of the two groups of patients were separated from each other, and the species diversity was different, which indicated that pneumoconiosis would lead to intestinal flora disorder, that the pathogenic bacteria or probiotics in the study group would increase or decrease, and that the intestinal flora might play a role in the occurrence and development of pneumoconiosis, which was consistent with the views of previous literature [28]. At the same time, the lung tissue damage in pneumoconiosis patients was more serious, which provides a basis for the previous literature to suggest that cardiopulmonary health could be used as a predictive index of intestinal flora.

Previous reports pointed out that species richness may be related to maintaining good health, and changes in microbial flora may be related to the increase of disease, so pneumoconiosis may inhibit or promote the growth of some flora. Actinomycetes are named for their radioactive colonies. Most of them had basal hyphae and aerial hyphae; some of them have no aerial hyphae. Most of them can produce conidia, forming cysts. The most important function of actinomycetes is the production of antibiotics [27]. In this study, the decrease in actinomycete abundance may lead to intestinal flora disorder, impaired immune function, the aggravation of inflammatory reaction, and increased risk in pneumoconiosis patients. Bifidobacterium is a type of Gram-positive, motionless, rod-shaped, sometimes branched at one end, strictly anaerobic bacteria. It widely exists in the digestive tract, vagina, and oral cavity of humans and animals [29]. Bifidobacteria are very important bacteria in the human body. They are a type of intestinal probiotic in the human body. The number of intestinal microbial bifidobacteria is very large, up to 109–1011/g feces, accounting for more than 85% of intestinal microorganisms. As a physiologically beneficial bacterium, it has many important physiological functions, such as biological barrier, nutrition, anti-tumor, immune enhancement, gastrointestinal function improvement, anti-aging, and so on [30]. In this study, we found that the abundance of Bifidobacterium pneumoconiosis in patients decreased, accompanied by physical injuries. Veillonella is a Gram-negative anaerobic Micrococcus with a diameter of 0.3–0.5 um and requires carbon dioxide for growth. The bacteria are strictly anaerobic and parasitic in the oral cavity, intestinal, and respiratory tracts of humans and animals. It can produce endotoxin; therefore, it plays a role in a variety of mixed infections which can be seen in the upper respiratory tract and intestinal tract. Veillonella can consume lactic acid and keep the body in a balanced state. The decrease in Veillonella in this study may lead to an increase in lactic acid in the body. Increased lactic acid will cause pneumoconiosis patients to feel weak and tired. The concept of Prevotella was first proposed by Haroun N. Shan in 1990. Prevotella is an absolutely anaerobic Gram-negative bacterium. Prevotella was once considered to be Bacteroides, but now they do not belong to the same genus because of their differences in genotype and phylogeny. Prevotella is an independent new mycelium now. Since then, many studies have shown that Prevotella has changed in many diseases, including bacterial vaginitis, asthma, autism in children with chronic obstructive pulmonary disease, rheumatoid arthritis, and so on. The amount of Prevotella in the gut of pneumoconiosis patients increased, but the reason for this is still unclear. At present, it can only be proven that Prevotella is closely related to pneumoconiosis, so further research is needed. However, this study found that pneumoconiosis could lead to intestinal flora disorder and identified the differential flora. At the same time, through correlation analysis, it was found that some differential metabolites were correlated with some differential flora, and the results are statistically significant. These results provided a new idea for the study of pneumoconiosis. However, this experiment is an observational study, and the exploration of the pathogenesis of pneumoconiosis is also speculated, which requires further study.

Intestinal flora is a real endocrine organ in the human body. Its molecules interact with the physiological functions of the human body and trigger reactions locally and in distal parts. A large number of metabolites drive the homeostasis between the human body and its flora. Intestinal microbiota affects the health of the host, especially the intestinal immune homeostasis and the intestinal immune response. Besides serving as a nutritional enhancer, L-tryptophan (Trp) plays a vital role in the balance between intestinal immune tolerance and maintenance of the intestinal microbiome. Recent discoveries have underscored that changes in the microbiota modulate the host immune system by modulating Trp metabolism [31]. Intestinal microflora regulates intestinal tryptophan metabolism and speed-limiting enzymes or tryptophan metabolites and causes tryptophan to exert its biological function through intestinal receptors. Intestinal tryptophan and its metabolites have a close interaction with intestinal flora in the aspects of the intestinal barrier, intestinal immune and endocrine functions, intestinal motility, etc. Other studies showed that the IBD state classifier based on metabonomics and metagenome is very accurate, and like most individual trends, it can be well extended to the independent validation queues [32]. Therefore, in the occurrence and development of pneumoconiosis, there may be a certain connection between differential metabolites and differential bacteria. While many of these changes likely resulted from physiological changes on the host side, the subset that could be confirmed to result from microbial activity would provide promising targets for microbiome-based pneumoconiosis diagnostics and therapies. Therefore, in future studies, we could further study the relationship between metabolites and bacteria. We plan to carry out animal intervention experiments first and then carry out crowd intervention experiments, for example, by adjusting the flora structure and observing the changes in metabolites. The mouse intervention experiment and crowd intervention experiment were used to verify the results of this study and further explore and analyze them to study whether intestinal flora intervention can have therapeutic effects on pneumoconiosis patients and provide basis for the treatment research of pneumoconiosis patients.

## 5. Conclusions

Diethanolamine, dipeptide, glutamic acid, tryptophan, phenylacryloyl tryptophan, formic acid, eicosapentaenoic acid, inosine, acamprosate, and phenacemide may be related to the occurrence and development of pneumoconiosis.

Metabolic pathways involved in different metabolites involved in pneumoconiosis include glycerol phospholipid metabolism, D-glutamine and D-glutamate metabolism, cyanate metabolism, aminoacyl tRNA biosynthesis, nitrogen metabolism, purine metabolism, and tryptophan metabolism. Among them, the change in aminoacyl tRNA biosynthesis was most obvious in pneumoconiosis.

Intestinal flora in pneumoconiosis patients is out of balance. In pneumoconiosis patients, the abundance of Veillinellaceae, Negativicutes, Actinobacteria, Bifidobacteriales, Selenomonadales, Actinobacteria, Bifidobacterium-uncultured, Bifidobacteriaceae, and Bifidobacterium decreased, while that of Prevotellaceae increased. These different bacteria may be related to the occurrence and development of pneumoconiosis, but they require further study.

## Figures and Tables

**Table 1:** PCR amplification program.

Location	Time	Cycle Number
98 °C	2 min	30 cycles
98 °C	30 s	
50 °C	30 s	
72 °C	60 s	
72 °C	5 min	
4 °C		

**Table 2:** Differential metabolites in positive ion mode.

Number	Ion Mode	Metabolites	Maximum Change Multiple	VIP	*p*	Metabolic Pathway
1	Positive ion mode	Diethanolamine	3.86	1.96	8.41534 × 10^−10^	Glycerol phospholipid metabolism
2	Positive ion mode	Dipeptide	6.69	2.56	1.21497 × 10^−5^	D- glutamine and D- glutamate metabolism
3	Positive ion mode	Glutamic acid	11.93	3.29	2.08556 × 10^−7^	Cyanamide metabolism
4	Positive ion mode	Tryptophan	7.39	4.32	9.41414 × 10^−9^	Aminoacyl tRNA biosynthesis
5	Positive ion mode	Phe-Try	2.41	4.91	2.3 × 10^−4^	Nitrogen metabolism

**Table 3:** Differential metabolites in anion mode.

Number	Ion Mode	Material Name	Maximum Change Multiple	VIP	*p* Value	Metabolic Pathway
1	Negative ion mode	Formic acid	2.300	1.32	4.4 × 10^−3^	Tryptophan metabolism
2	Negative ion mode	Eicosapentaenoic acid	2.566	1.09	1.6 × 10^−2^	
3	Negative ion mode	Acanoic acid	3.986	1.51	2.05937 × 10^−7^	
4	Negative ion mode	Inosine	2.949	3.28	5.3 × 10^−4^	Purine metabolism
5	Negative ion mode	Phenylacetylurea	2.906	2.28	1.6 × 10^−3^	

**Table 4:** Differential metabolite characterization.

Metabolites	Molecular Formula	Change	Existence and Source	Function
Glutamic acid	C_5_H_9_NO_4_	Rise	Cereal protein and animal brain	Participates in many important chemical reactions in animal, plant and microorganism.
Diethanolamine	C_4_H_11_NO_2_	Rise	-	Gas purifier; Synthetic drugs and raw materials for organic synthesis; synthesis of glycerol phosphatide
Tryptophan	C_11_H_12_N_2_O_2_	Rise	-	Important precursors of auxin biosynthesis in plants. Important neurotransmitter in the human body is the precursor of 5- erotonin. Participates in the renewal of plasma protein in animals; promotes riboflavin to play a role; contributes to the synthesis of nicotinic acid and heme
Eicosapentaenoic Acid	C_20_H_30_O_2_	Rise	Food (sardine, etc.)	Used for health care
Inosine	C_10_H_12_N_4_O_5_	Rise	Composed of hypoxanthine and ribose	Used as coenzyme drugs; improves the level of ATP; an auxiliary liver-protecting drug
Acamprosate	C_5_H_11_NO_4_S	Rise	Derivatives of taurine	Treatment of alcohol addiction
Phenylacetylurea	C_9_H_10_N_2_O_2_	Rise	-	Synthesis of antituberculosis drugs thiourea and sulfonamide; rodenticide; analytical reagent for quantitative determination of chromium and identification of aldehydes and ketones

**Table 5:** Quality statistics of intestinal microbiological sequencing data of two groups.

Group	Samples	PE Reads	Clean Tags	Effective Tags	AvgLen (bp)	GC (%)	Effective (%)
Control group	A1	79,736	76,692	56,155	416	53.98	70.43
	A2	79,941	77,337	59,944	409	53.63	74.99
	A3	79,998	77,102	52,758	410	52.83	65.97
	A4	79,977	77,050	61,366	409	52.59	76.74
	A5	79,962	75,610	63,058	408	54.06	79.02
	A6	79,800	76,476	59,411	412	53.59	74.08
	A7	80,200	75,698	61,292	412	53.67	76.8
	A8	79,812	76,211	62,648	427	54.28	78.37
	A9	79,939	76,432	60,284	417	54.51	75.49
	A10	79,856	76,639	58,172	417	55.46	72.82
	A11	79,890	74,239	64,256	412	54.15	80.51
	A12	79,815	75,760	59,902	421	54.74	74.96
	A13	79,911	75,448	58,943	408	54.32	73.78
	A14	79,888	76,845	67,853	411	57.8	84.72
	A15	80,088	77,226	62,664	411	57.55	78.57
Study group	B1	79,755	76,680	62,322	421	51.5	77.95
	B2	79,953	77,018	58,276	410	52.18	72.81
	B3	80,035	77,086	55,139	410	53.66	69.14
	B4	80,369	77,660	61,955	410	52.56	77.09
	B5	79,798	77,466	63,235	416	54.13	79.24
	B6	79,962	75,041	60,358	413	53.98	75.48
	B7	80,091	75,911	64,254	413	53.85	80.23
	B8	79,918	77,444	59,203	410	53.33	74.08
	B9	80,096	76,968	68,829	417	53.96	85.93
	B10	79,944	76,595	68,422	417	53.88	85.59
	B11	79,867	76,712	70,834	418	54.11	88.69
	B12	80,313	76,260	63,738	418	54.83	79.36
	B13	79,371	75,726	55,646	411	54.57	70.11
	B14	79,899	75,761	62,840	416	52.43	78.65
	B15	80,084	76,874	67,742	407	54.44	84.59

**Table 6:** The statistical table of species in different grades of samples.

Classification Level	Control Group	Study Group
Phylum	8.00 ± 0.66	8.47 ± 0.52
Class	13.60 ± 0.83	14.40 ± 0.51
Order	26.80 ± 1.21	27.60 ± 0.99
Family	47.20 ± 2.65	47.87 ± 1.99
Genus	112.27 ± 5.85	117.93 ± 3.43
Species	126.27 ± 6.85	132.33 ± 5.354

**Table 7:** Alpha diversity index statistics.

Index	Control Group	Study Group
ACE	233.80 ± 20.45	238.87 ± 11.25 *
Chao1	133.78 ± 18.89	239.49 ± 13.33 *
Shannon	0.27 ± 0.15	0.18 ± 0.08 *
Simpson	2.27 ± 0.65	2.53 ± 0.56 *

* There are statistical differences.

**Table 8:** Morphological characterization of differential flora.

Flora	Form	Size	Spore Flagella	Gram Stain	Main Habitat
Veillonellaceae	Globular	0.3–2.6 μm	No spores, no flagellum.	Negative	Anaerobic
Actinobacteria	Radial	The mycelial diameter is 1 μm	Most of them produce conidia, and some form spores and flagella.	Positive	Anaerobic or facultative anaerobic
Bifidobacterium	Rod shape	0.5–1.3 μm × 1.5–8 μm	No spores, no flagellum.	Positive	Strict anaerobic
Prevotellaceae	Rod shape	-	No spores, no flagellum.	Negative	Strict anaerobic

**Table 9:** Other characterization of differential bacteria.

Flora	Fermentation Metabolism	Major Metabolites of Fermentation Metabolism	Pathogenicity	Distribution
Veillonellaceae	Pyruvic acid, lactic acid, malic acid, pyruvic acid and oxalic acid	Acid and gas production	When mixed infection, endotoxin can be produced	The gut of warm-blooded animals (such as ruminants, rodents, and pigs)
Actinobacteria	Sugar, starch, organic acid, cellulose, hemicellulose, etc	A wide variety of antibiotics	Can cause actinomycosis	Widely distributed in soil
Bifidobacterium	Sugar	Lactic acid, acetic acid	-	Human and animal habitats, such as the digestive tract, vagina, and oral cavity
Prevotellaceae	Sugar	Acetic acid and succinic acid and a small amount of isobutyric acid, isovalerate, and lactic acid	Conditional treatment	Oral cavity, upper respiratory tract, urogenital tract

## Data Availability

All data generated or analyzed during this study are included in this published article. All raw and analyzed data are accessible.
